# Computational Investigation of Tuning the Electron-Donating Ability in Metal-Free Organic Dyes Featuring an Azobenzene Spacer for Dye-Sensitized Solar Cells

**DOI:** 10.3390/nano9010119

**Published:** 2019-01-18

**Authors:** Md Al Mamunur Rashid, Dini Hayati, Kyungwon Kwak, Jongin Hong

**Affiliations:** 1 Center for Molecular Spectroscopy and Dynamics, Institute for Basic Science (IBS) & Department of Chemistry, Korea University, Seoul 02841, Korea; ndcmamun@korea.ac.kr; 2 Department of Chemistry, Chung-Ang University, Seoul 06974, Korea; dinihayati300194@gmail.com

**Keywords:** Dye-sensitized solar cell (DSSC), time-dependent density functional theory (TDDFT), donor–π-conjugated spacer–acceptor (D–π–A) azobenzene-based dyes, intramolecular charge transfer

## Abstract

A series of donor–π-conjugated spacer–acceptor (D–π–A) organic dyes featuring an azobenzene spacer were designed as chromic dyes and investigated computationally. The electron-donating strength was modified by introducing electron-donating units to the donor side. In particular, the *trans*–*cis* isomerization of the azobenzene-based dyes and its effect on the optical and electronic properties were further scrutinized. In both *trans* and *cis* conformers, a gradual increase in electron-donating strength promoted the natural charge separation between donor and acceptor moieties, thereby allowing the absorption of a longer wavelength of visible light. Importantly, the conformational change of the azobenzene bridge resulted in different absorption spectra and light-harvesting properties. The azobenzene-based dyes will open up a new research path for chromic dye-sensitized solar cells.

## 1. Introduction

The efficient usage of solar energy is considered as the most promising way to meet an everlasting global demand for energy. Dye-sensitized solar cells (DSSCs) attracted significant attention as a substitute for commercialized crystalline and thin-film Si solar cells. In public sectors, the DSSCs hold particular promise on flat and curved building skins in building-integrated photovoltaics (BIPVs) because of their transparency and aesthetic value. To date, numerous efforts on device physics, material innovation, and commercialization were made to achieve high performance and long-term fidelity of the DSSCs [1,2,3]. The photosensitizer is one of the key components for both power conversion efficiency and stability of the cell. Recently, metal-free organic photosensitizers featuring a donor (D)–π conjugation bridge (π)–acceptor (A) structure received increasing interest as a viable alternative to conventional ruthenium-based dyes [4,5,6]. Major advantages of the organic dyes are high molar extinction coefficients, easy synthesis and purification, and tunability. Many groups focused on their molecular architecture by varying donor, π-bridge, and acceptor units theoretically and experimentally. For example, triphenylamine (TPA) and cyanoacrylic acid moieties were found as valuable electron donor and acceptor units, respectively. Various π-bridges including alkyne, aromatic, or heteroaromatic residues were investigated to modify the optical properties of the photosensitizers and to control their intramolecular charge transfer (ICT) properties related to electron injection into the semiconductor (e.g., TiO_2_, ZnO). The efficiencies of the DSSCs based on these photosensitizers gradually increased over the past decade and reached those of ruthenium-based DSSCs [7]. Regrettably, these molecules are not responsive to different local or external stimuli including light and heat. Thus, if a moiety sensitive to one or more external stimuli is introduced to the metal-free organic dyes, new functional dyes can be explored for the DSSCs.

With the advent of high-performance computing, computational science emerged as a viable and powerful approach to find new functional molecules prior to expensive and time-consuming synthesis. Theoretical calculations can provide a molecular-level understanding of their geometric and physical properties. Density functional theory (DFT) and time-dependent DFT (TDDFT) were extensively used to investigate the electronic and optical properties in the ground and excited states of virtual molecules in the development of DSSCs [8,9]. Notably, the results of TDDFT calculations are comparable to those of expensive high-level calculations, such as configuration interaction single (CIS), coupled-cluster singles and doubles (CC2), and multireference perturbation theory (MRPT), although the TDDFT calculations strongly depend on the choice of an exchange–correlation functional. Therefore, the theoretical predictions based on the DFT calculations offer a real promise of being able to complement experiments in the DSSCs [10,11,12]. 

Among environmentally responsive molecules, azobenzene and its derivatives containing an azo moiety (–N=N–) have the ability to alter their geometries via photochemical or thermal *trans*–*cis* (*E*⇌*Z*) isomerization [13,14]. Their reversible *trans*–*cis* isomerization was widely adopted to develop various molecular switches and machines. Recently, a few groups reported azobenzene-based dyes as photosensitizers for the DSSCs, even though they did not show excellent power conversion efficiency (PCE) in the cells [15,16,17,18]. Unfortunately, the effect of *trans*–*cis* isomerization on DSSC performance is not widely investigated. Recently, Pang and co-workers reported the torsional effects on the optical properties of three azobenzene derivatives in vacuum and dimethyl sulfoxide (DMSO) [19]. However, they did not investigate their structural and electronic properties in detail. Novir and Hashemianzadeh investigated the geometrical and electronic properties of seven aminoazobenzene dyes with different donating groups and different positions of carboxylic acid, and determined the parameters affecting the efficiency of DSSC [20]. Regrettably, the replacement of the donating groups did not provide any significant change in the optical properties of the azo dyes. In our study, we further investigated the *trans*–*cis* isomerization of various TPA-based organic dyes, upon changing the substituents on the TPA moiety featuring the azobenzene bridge, using DFT and TDDFT calculations. Natural bond orbital (NBO) analysis was also carried out to quantitatively determine the amount of charge transfer from electron donors to electron acceptors of both *trans* and *cis* isomers. In addition, the interrelationship between geometric parameters and optical properties was discussed in terms of chromic dyes for the DSSCs.

## 2. Computational Details 

All calculations were performed with Gaussian 09 [21] following the ground-state optimization of the geometries with DFT, and the determination of the vertical electronic excitation energies and oscillator strengths for the lowest 20 transitions by means of TDDFT using the ground-state optimization structures. For DFT calculations, the popular B3LYP functional was used for optimization, which was previously justified for this type of calculation [22], comparing different parameters such as bond lengths and dihedral angles [23]. For TDDFT calculations, we used the range-separated Coulomb-attenuating CAM-B3LYP [24] functional, which is suitable for the intramolecular charge transfer type of excitation used. Berardo studied the excited state relaxation in naked and hydrated TiO_2_ nanoparticles using CAM-B3LYP, BHLYP, and B3LYP functionals, and found that CAM-B3LYP showed consistency with coupled-cluster theory, whereas B3LYP predicted different chemical characteristics along with underestimating the vertical excitations, as well as creating a charge transfer (CT) problem during excited-state relaxation for certain particles even in the hydrated particles [25]. Yin et al. used B3LYP, PBE0, CAM-B3LYP, M06-2X, M06, LC-ωPBE, and ωB97X-D for excited-state calculations of azobenzene-based photoswitches in order to ensure the reliability of the selected functionals, where CAM-B3LYP exhibited good performance reproducing the examined absorption spectra [26]. We selected the 6-311G(d, p) basis set for ground-state optimization, as well as for the TDDFT calculations. As the presence of solvent effects is crucial to explain the absorption spectra of the sensitizers, the effect of solvation (acetonitrile, e = 35.688) was introduced to excited-state calculation through a self-consistent reaction field (SCRF) using Tomasi’s polarizable continuum model (PCM) [27]. Natural bond orbital (NBO) analysis was conducted by calculating the orbital populations with the B3LYP/6-311G(d, p) method for the ground state and the CAM-B3LYP/6-311G(d, p) method for the excited state using the NBO 5.0 program. Vibrational frequency analysis was also performed with the same level of theory, where no imaginary frequency was found, confirming that the optimized structures are true minima on the potential energy surface.

## 3. Results and Discussions

**Figure 1:**
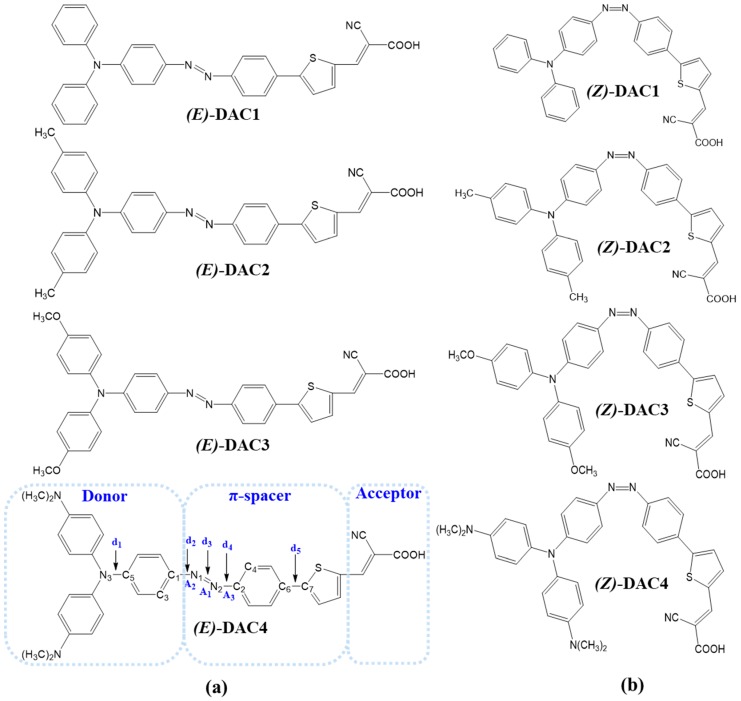
Molecular structure of the dyes: (**a**) *trans* dyes, and (**b**) *cis* dyes. The sky-blue dotted blocks represent the donor, π-spacer, and acceptor; d represents the bond distance and A denotes the dihedral angle.

In this computational study, we designed D–π–A organic dyes containing each donor unit, triphenylamine, 4,4’-dimethyltriphenylamine, 4,4’-dimethoxytriphenylamine, and bis(4-dimethylaminophenyl)-phenyl-amine). Substituents in which the *para* position of the terminal phenyl group was replaced are known as *para*-directors. The donor units with bulky substitutions are useful for longer electron lifetime, higher V*_oc_* values, and better suppression of back electron transfer for the DSSCs [28]. The cyanoacrylic acid group was chosen as the electron acceptor. The azobenzene and thiophene linker group was adopted as the extended π-conjugated bridge to improve the photovoltaic properties of the organic dyes [29]. In particular, the azo group has reversible *trans*–*cis* isomerization and, thus, its introduction to the π-conjugation backbone allows for geometrical change due to external stimuli, such as light and heat. The molecular structures of the D–π–A dyes are illustrated in Figure 1. Figure 1a,b represent *trans* and *cis* structures, respectively. The *trans* structures are named (*E*)-DAC1, (*E*)-DAC2, (*E*)-DAC3, and (*E*)-DAC4, and the *cis* structures are named (*Z*)-DAC1, (*Z*)-DAC2, (*Z*)-DAC3, and (*Z*)-DAC4, in ascending order of electron-donating strength.

**Figure 2:**
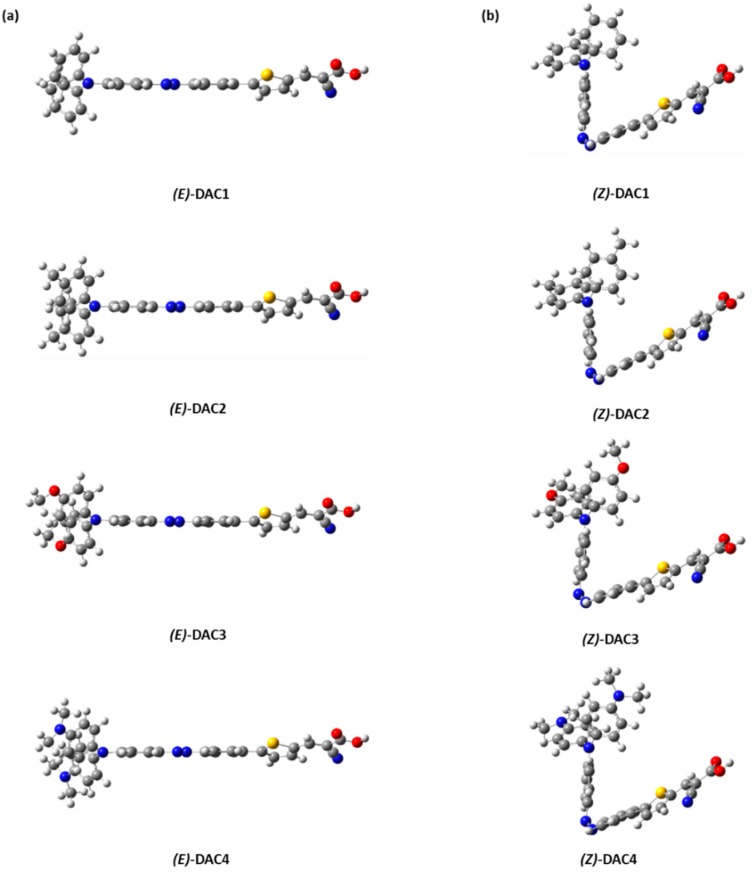
Optimized geometries of the (**a**) *trans* and (**b**) *cis* dyes.

Figure 2a,b show the optimized geometries of both *trans* and *cis* geometries at the B3LYP/6-311G(d, p) level, respectively. The *trans* dyes were fully conjugated throughout the donor, π-bridge, and acceptor groups, whilst the *cis* dyes were three-dimensionally distorted with a dihedral angle (C_1_N_1_N_2_C_2_) of ~12°. Because of the strong π-conjugated effect [30], the planar *trans* dyes suppress the rotational disorder and transfer more charges from donor to acceptor than the distorted *cis* dyes. Unfortunately, the dihedral angles between thiophene and adjacent benzene units were ~23°, presumably due to steric hindrance between the hydrogens of thiophene and benzene moieties. TPA units had a distorted three-dimensional structure with a dihedral angle between the phenyl rings of ~50°, due to the internal steric hindrance. To find the relationship between geometric properties and electron-donating strength of the dyes, the selected bond lengths and the dihedral angles from the optimized structures are summarized in Table 1. The five bond lengths are denoted as d_1_ d_2_, d_3_, d_4_, and d_5_, and the three dihedral angles are symbolized as A_1_, A_2_, and A_3_ (Figure 1). The calculated bond lengths were between values of single and double bonds (i.e., C–C: 1.530 Å, C=C: 1.339 Å [31], N–C: 1.471 Å [32], N=C: 1.273 Å [33], and N=N: 1.247 Å [34]). This indicates that charges were extensively delocalized throughout the entire molecules. Interestingly, the N=N bond (d_3_) length of the *trans* dyes was longer than that of the *cis* dyes, whilst both C–N bonds (d_2_ and d_4_) next to the N=N bond of the former were shorter than those of the latter. As the electron-donating strength of the donor group increased, the C–N distances decreased, but the N=N distances increased in both *trans* and *cis* dyes. This implies that the electron-donating strength directly affects the geometric features, which are related to the electronic structures, charge transfer, and optical properties. In addition, even with the large displacement from the *trans* form to the *cis* form, we can still observe the alternation of the bond lengths as a function of electron-donating strength. We think that the π-conjugation in the azobenzene group can be maintained during the *trans*–*cis* isomerization, even though the *cis* isomers have distorted non-planar structure around azo group. Unfortunately, the C–C bond length (d_5_) was not sensitive to the electron-donating strength in both the *trans* and *cis* dyes because the π-conjugation was broken between the thiophene and the adjacent benzene moieties.

The NBO analysis of the ground state was performed to determine the distribution of charge on the overall dye molecules and to estimate the extent of an intramolecular charge transfer (ICT). The NBO charges for donor, π-spacer, and acceptor are denoted as q^Donor^, q^π-spacer^, and q^Acceptor^, respectively, and they are summarized in Table 2. A positive NBO value represents that TPA or its derivatives are electron donors. A negative one indicates that the cyanoacrylic acid acts as the electron acceptor. It should be noted that the π-spacer (azobenzene–thiophene) behaves like the electron acceptor since it shows negative NBO charge. The biggest charge variance between natural charges on the donor (q^Donor^) and acceptor (q^Acceptor^) groups is represented in a ∆q^D−A^ value. The *cis* (*Z*) dyes had lower ∆q^D−A values^ than the *trans* (*E*) dyes; thus, the *trans* structure of the π-spacer was more favorable for ICT than the *cis* structure. Higher electron-donating strength also results in higher ∆q^D−A^ value. In addition, the q^π-spacer^ of *trans* dyes was almost double that of *cis* dyes. We confirm that the strong electron-donating group promoted the natural charge separation between donor and acceptor groups in our D–π–A dyes, and the ICT ability was sensitive to the conformational change in the π-spacer. To describe the amount of charge separation between the donor and the acceptor groups, second-order perturbation theory (SOPT) analysis was also performed within the NBO basis represented in Table S1 (Supplementary Materials) [35]. It is shown that, with increasing the donor strength, the conjugative interaction energy (*∆E*^2^) also increased from DAC1 to DAC4. Figure S1 (Supplementary Materials) shows the *∆E*^2^ between the donor and the π-spacer (D–π) as a function of C–N bond lengths (d_1_, d_2_, and d_4_). It is shown that DAC4 had the highest *∆E*^2^ with the shortest C–N bond lengths in both *trans* and *cis* dyes, whereas (*E*)-DAC4 showed higher *∆E*^2^ than (*Z*)-DAC4. This indicates that electronic charge separation can be a simple function of the C–N bond lengths, because the electronic conjugation through the organic molecules depends on both the donor strength and electronic charge transfer. Furthermore, it is shown that the *∆E*^2^ of *trans* dyes was higher than that of *cis* dyes in the case of π(C_1_=C_2_) to π*(N_1_=N_2_), which suggests that the conformational changes of the dyes also affect the *∆E*^2^.

**Figure 3:**
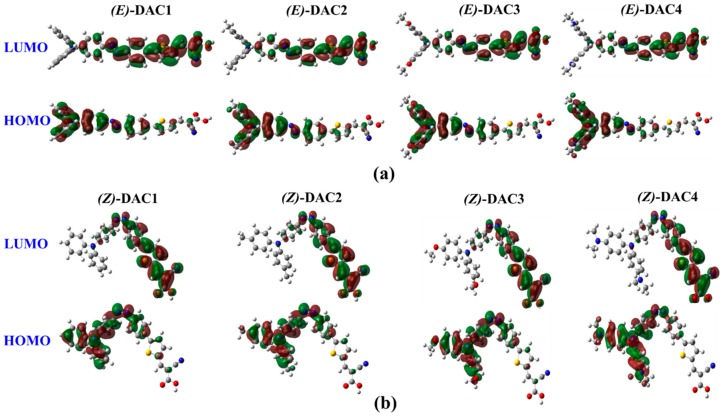
Frontier molecular orbitals of (**a**) *trans* dyes and (**b**) *cis* dyes.

The ICT likely occurs from electron donor to electron acceptor under light illumination. In an efficient light-harvesting sensitizer, the highest occupied molecular orbital (HOMO) and lowest unoccupied molecular orbital (LOMO) of the whole system are commonly dominated by donor and acceptor groups, respectively [36]. The frontier molecular orbitals are important indicators to predict the optical and electronic properties of the photosensitizer. The electronic density distributions of the HOMO and LUMO of *trans* and *cis* dyes are illustrated in Figure 3. Figure 3a,b represent the electron densities of the frontier molecular orbitals of *trans* (*E*) and *cis* (*Z*) dyes, respectively. The HOMO and LUMO show π and π* characteristics, respectively. The electron densities of HOMOs are mainly localized on the donor unit, whereas those of LUMOs are extended along the π-spacer to the cyanoacrylic acid unit. This ICT characteristic causes a net electron transfer from the donor group to the anchoring group to the TiO_2_ surface. It can be assumed that the position of the LUMO close to the cyanoacrylic acid would enhance the orbital overlap with the Ti 3*d* orbitals; thus, excited electrons could be easily injected into TiO_2_ via the anchoring unit. The frontier molecular orbitals suggest that all the dyes provide large ICT and strong electronic coupling with TiO_2_ surface. It is also observed that the *cis*–*trans* conformation did not affect the HOMO–LUMO electron distribution much. This indicates that azobenzene–thiophene can be a good π-spacer for ICT under illumination. Moreover, the ICT characteristic maintained even with the clear structural change. Accordingly, we expect that both the *trans* and *cis* forms work as the photosensitizer for DSSCs.

**Figure 4:**
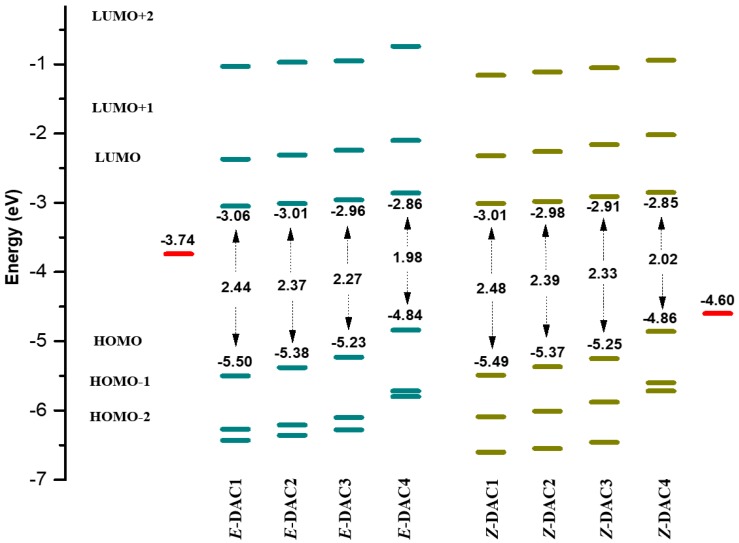
Schematic energy diagrams of dyes relative to the conduction band of TiO_2_ and electrolyte (I^−^/I_3_^−^) redox potential.

Figure 4 shows the molecular orbital energies of all the D–π–A dyes from HOMO−2 to LUMO+2 levels calculated with B3LYP/6-311G(d, p) with the PCM model. The measured HOMO values of the dyes show that the HOMO energy levels of all the dyes were lower than the redox potential of I^−^/I_3_^−^ (−4.60 eV) [37], which ensures restoring of electrons from the electrolyte. Similarly, the LUMO levels of the *cis* and *trans* dyes were higher than the TiO_2_ conduction band (−3.74 eV) [38], which indicates the ability of the dyes to inject electrons into TiO_2_. The HOMO–LUMO energy gaps are summarized in Table 3. In both the *trans* and *cis* dyes, the stronger donating strength of the donor group (i.e., DAC1 < DAC2 < DAC3 < DAC4) allowed for decreasing the HOMO–LUMO gaps and, thus, absorbing a longer wavelength of visible light. This coincides with the result of the NBO analysis. It is known that low-bandgap dyes show high power conversion efficiency in the DSSCs [39]. Chemical hardness (η), which is considered as the resistance to intramolecular charge transfer in the solar cells, was evaluated for all the dyes, and the results are summarized in Table 3 [40]. The chemical hardness (η) can be obtained using the Koopmans’s theorem as follows:

(1) $$\eta =\frac{\left[IP-EA\right]}{2}\approx \frac{\left[E_{L}-E_{H}\right]}{2}$$

where IP and EA are the ionization potential and electron affinity, respectively. It is shown that *trans* dyes have lower η than *cis* dyes, which means that the *trans* dyes show better efficiency for DSSCs.

It should be noted that the *trans* dyes are more effective in absorbing visible light than *cis* dyes. Accordingly, the energy bandgap of the azobenzene-based dyes can be tuned by means of introducing a suitable electron-donating group, as well as twisting the molecular conformation. The energy difference between *trans* and *cis* conformers is also presented in Table 3. Irrespective of the change in the electron-donating strength, the total energy difference remained almost same. This means that the relative population of *trans* and *cis* conformers was independent of the choice of electron-donating group. Another important factor related to the DSSC performance is the dipole moment of the dyes, which can shift the conduction band of the wide-bandgap semiconductor (e.g., TiO_2_, ZnO) [41]. Stronger electron-donating ability also results in a larger dipole moment of the dyes. A large dipole moment can increase the distance between the charge centers, thus resulting in enhanced electron delocalization. The *trans* dyes have a larger dipole moment than *cis* dyes. The lower dipole moment of the *cis* structure compared to the *trans* structure decreases the bond polarity; thus, the dipole moment vectors of the bonds cancel each other out [42].

**Figure 5:**
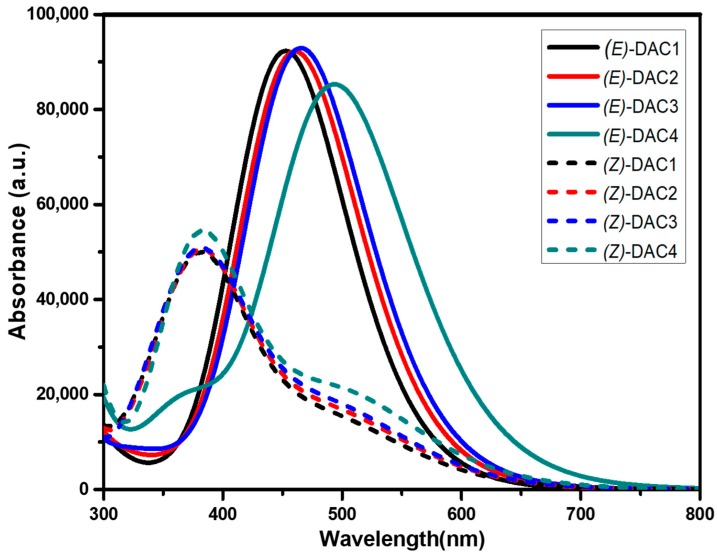
Ultraviolet–visible light (UV–Vis) absorption spectra of the dyes. The normal lines represent the *trans* dyes and the dotted lines represent the *cis* dyes.

Maximum absorption wavelengths (λ_max_), vertical excitation energies (E_ex_), oscillator strength (*f*), light-harvesting efficiencies (LHE), and molecular orbital excitations for the most relevant transitions of the electronic absorption bands were calculated, and the results are summarized in Table 4. The simulated absorption spectra of *trans* and *cis* dyes in acetonitrile from the TDDFT calculations are also shown in Figure 5. The solid lines represent the *trans* (*E*) dyes, whereas the dotted lines represent the *cis* (*Z*) dyes. The *trans* dyes show stronger light absorption in the visible regime than the *cis* dyes. The bathochromic shift of λ_max_ values was related to the electron-donating strength of the donor group (i.e., DAC1 < DAC2 < DAC3 < DAC4), and this shift was more favorable for the *trans* dyes. This indicates that the *trans* dyes had better light-harvesting properties than the *cis* dyes. Interestingly, two absorption bands were observed in the *cis* dyes. The strong absorption band around 370 nm was likely due to the π–π* transition and the weak one around 500 nm was attributed to n–π* transition. It should be noted that this spectral difference between *trans* and *cis* isomers will result in different colors in the DSSCs. 

From Table 4, the transition from HOMO to LUMO corresponding the π–π* transition was the main contribution to the lowest electronic excitation in the *trans* dyes, although the transitions from other occupied orbitals somewhat contributed. However, the transition from HOMO−1 to LUMO representing the π–π* transition mostly contributed to the strong absorption in the *cis* dyes. The weak absorption in the *cis* dyes was related to all the transitions of the occupied orbitals corresponding to the n–π* transition, and this was due to the presence of unshared electron pairs of nitrogen atoms. It should be noted that such n–π* transition is not allowed in the *trans* dyes because of the symmetry rules [13]. The coplanar structure of the azobenzene unit in the *trans* dyes forbids the n–π* transition, whilst the n–π* transition in the *cis* dyes results from the interaction between the azo bond (N=N) and the π-conjugation system. The performance of a cell converting solar energy to electric efficiency is dependent on light-harvesting efficiency (LHE), that is, light absorbance by the dye-sensitized TiO_2_ film. LHE can be calculated using the following equation:

(2) $$LHE=1-\;10^{-A}=1-10^{-f}$$

where *f* is the oscillator strength of the dye corresponding to the absorption maximum (λ_max_). Generally, greater LHE values, due to higher oscillator strength, increase the light-capturing ability and improve the efficiency of the DSSC [43]. In Table 4, it can be seen that the oscillator strength (*f*) of *trans* dyes was higher (~2.0) that that of *cis* dyes (<1.0). Eventually, the LHE of *trans* dyes was higher than that of *cis* dyes, suggesting that *trans* dyes have more light-harvesting capability than *cis* dyes. In the case of *cis* dyes, it was observed that, for the π–π* transition, the LHE values were higher than those for the n–π* transition, which indicates that the π-π* transition is favorable for LHE for both the trans and cis dyes. It is also observed that changing the donor moiety in both the *trans* and *cis* dyes did not affect the oscillator strength or the LHE, concluding that the light-harvesting property is influenced by the conformational change of the structures. It was also observed that both the oscillator strength and the LHE had great influence on the conformational change of the azobenzene bridge, but not on the electron-donating strength in the donor group.

**Figure 6:**
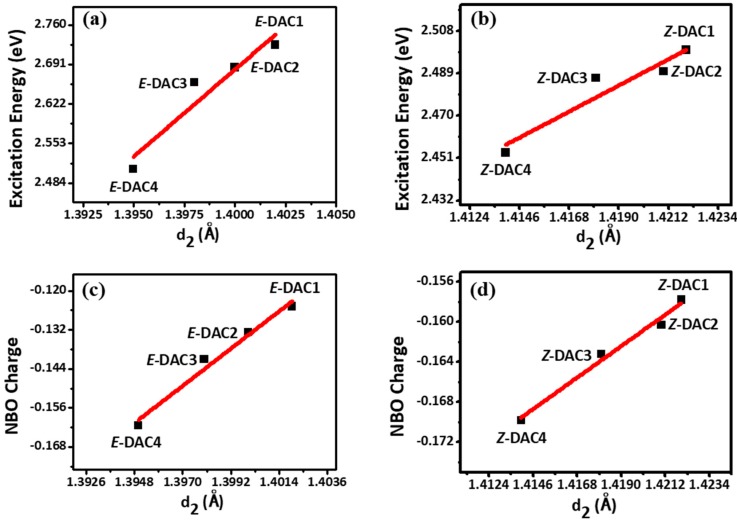
Plots of the excitation energy vs. distance d_2_ for (**a**) *trans* dyes and (**b**) *cis* dyes; natural bond orbital (NBO) charges of π-spacer moiety vs. distance d_2_ for (**c**) *trans* dyes and (**d**) *cis* dyes.

The electronic and optical properties were closely correlated with the geometric variables, such as the bond length. Recently, Seo et al. reported that the C–N bond length in the triphenylamine-based organic dyes was most susceptible to the donor strength [44]. In this respect, the vertical excitation energy and NBO charges were plotted as a function of C–N bond lengths (e.g., d_1_, d_2_, and d_4_). Figure 6a,b show vertical excitation energies as a function of d_2_ distance of the *trans* and *cis* dyes, respectively. Both (*E*)-DAC4 and (*Z*)-DAC4 exhibited the minimum excitation energy value and shortest d_2_ values due to the strongest donor. This is consistent with the longest wavelength absorption properties. As the donor strength increased from DAC1 to DAC4, the vertical excitation energy and d_2_ distance of both the *trans* and *cis* dyes decreased. Notably, the two variables exhibited a linear relationship in this study. Other C–N bond lengths (d_1_ and d_4_) also had the same trends with respect to the excitation energy, as shown in Figure S2 (Supplementary Materials). Figure 6c,d illustrate the NBO charges of the π-spacer as a function of d_2_ distance of the *trans* and *cis* dyes, respectively. Molecules with a shorter d_2_ distance showed more negative NBO charges. The two variables exhibited a linear dependence. Both d_1_ and d_4_ distances showed the same trends with respect to the NBO charge (Figure S3, Supplementary Materials). In addition, as all the C–N bond lengths were shorter, the NBO charges of the donor and acceptor were more positive and more negative, respectively (Figures S4 and S5, Supplementary Materials). These NBO charges were also linearly dependent on the C–N bond distances. Accordingly, the linear tendency of the optical and electronic charge properties with the C–N bond distances could be established in the azobenzene-based organic dyes. 

## 4. Conclusions

In summary, four D–π–A organic dyes featuring an azobenzene spacer were designed, and the effects of the substituted donor groups and the conformational change in the π-spacer on photovoltaic properties were computationally investigated. The DFT and TDDFT calculations showed that a gradual increase in the electron-donating strength promoted the natural charge separation between the donor and acceptor moieties, thus allowing for better light-harvesting properties in both the *trans* and *cis* dyes. The *trans*–*cis* isomerization of the azobenzene unit resulted in different absorption spectra and light-harvesting properties. In addition, the bond distances between nitrogen and carbon atoms in the donor and π-spacer parts were strongly correlated with the electronic and optical properties; thus, such geometric parameters could be suggested as simple and reliable descriptors of metal-free organic dyes for the DSSCs. We hope that this paper will encourage such azobenzene-based organic dyes to be used for further development of chromic dye-sensitized solar cells.

## Figures and Tables

**Table 1:** Selected bond lengths (Å) and dihedral angles (°) of the dyes.

	(*E*)-DAC1	(*E*)-DAC2	(*E*)-DAC3	(*E*)-DAC4	(*Z*)-DAC1	(*Z*)-DAC2	(*Z*)-DAC3	(*Z*)-DAC4
**d_1_(C_5_-N_3_)**	1.404	1.401	1.397	1.392	1.405	1.403	1.397	1.392
**d_2_(C_1_-N_1_)**	1.402	1.400	1.398	1.395	1.422	1.421	1.418	1.414
**d_3_(N_1_=N_2_)**	1.261	1.262	1.263	1.264	1.247	1.248	1.249	1.251
**d_4_(C_2_-N_2_)**	1.412	1.411	1.410	1.409	1.426	1.425	1.424	1.422
**d_5_(C_6_-C_7_)**	1.461	1.461	1.461	1.461	1.461	1.461	1.461	1.461
**A_1_(C_1_N_1_N_2_C_2_)**	179.351	179.371	179.219	179.652	−12.214	−12.376	−12.417	−12.694
**A_2_(C_3_C_1_N_1_N_2_)**	−2.036	−1.903	−1.512	−1.765	−37.821	−36.679	−34.342	−30.862
**A_3_(N_1_N_2_C_2_C_4_)**	−1.562	−1.322	−0.804	−0.722	−54.091	−54.507	−55.525	−56.612

**Table 2:** Natural bond orbital (NBO) analysis of metal-free organic dyes in the ground state where q^Donor^, q^π-spacer^, and q^Acceptor^ denote the total amount of natural charge on the donor group, π-spacer, and the acceptor group, respectively. ∆q^D−A^ represents the charge variance between natural charges on the donor and the acceptor group.

Dyes	q^Donor^	q^π-spacer^	q^Acceptor^	∆q^D−A^
(*E*)-DAC1	0.2827	−0.1249	−0.1578	0.4405
(*E*)-DAC2	0.2931	−0.1328	−0.1603	0.4534
(*E*)-DAC3	0.3044	−0.1411	−0.1632	0.4676
(*E*)-DAC4	0.3313	−0.1615	−0.1698	0.5011
(*Z*)-DAC1	0.2128	−0.0529	−0.1598	0.3726
(*Z*)-DAC2	0.2234	−0.0607	−0.1626	0.386
(*Z*)-DAC3	0.2384	−0.0726	−0.1657	0.4041
(*Z*)-DAC4	0.2677	−0.0936	−0.1741	0.4418

**Table 3:** Calculated highest occupied molecular orbital (HOMO), lowest occupied molecular orbital (LUMO), band energy gap (E_gap_), ionization potential (IP), electron affinity (EA), chemical hardness (η), dipole moment (µ), and Gibbs energy difference between *cis* and *trans* conformers of the dyes under the B3LYP/6-311G(d, p) basis set. All energies are given in eV.

Dyes	HOMO	LUMO	E_gap_	IP	EA	η	Dipole, µ (Debye)	∆E (E_cis_ − E_trans_)
(*E*)-DAC1	−5.50	−3.06	2.44	5.32	3.26	1.03	8.564	0.71
(*Z*)-DAC1	−5.49	−3.01	2.48	5.30	3.16	1.07	6.524	
(*E*)-DAC2	−5.38	−3.01	2.37	5.22	3.25	0.99	9.765	0.71
(*Z*)-DAC2	−5.37	−2.98	2.39	5.20	3.15	1.03	7.319	
(*E*)-DAC3	−5.23	−2.96	2.27	5.11	3.24	0.94	12.228	0.72
(*Z*)-DAC3	−5.25	−2.91	2.34	5.11	3.14	0.99	9.497	
(*E*)-DAC4	−4.84	−2.86	1.98	4.74	3.22	0.76	13.981	0.71
(*Z*)-DAC4	−4.86	−2.85	2.01	4.74	3.13	0.81	11.036	

**Table 4:** Maximum absorption wavelengths (λ_max_), vertical excitation energies (E_ex_), oscillator strength (*f*), light-harvesting efficiency (LHE), and transition assignment for the single transition of the absorption peak in the visible and near-ultraviolet region of the dyes using the time-dependent density functional theory (TDDFT) CAM-B3LYP/6-311G(d, p) method. All energies are given in eV.

Dyes	Excited State	E_ex_ (λ_max_)	*f*	LHE	Transition Assignment			
(*E*)-DAC1	π → π*	2.725 (455.05)	1.721	0.981	H−0→L+0 44%	H−1→L+0 14%	H−0→L+1 14%	
(*E*)-DAC2	π → π*	2.685 (461.75)	2.167	0.993	H−0→L+0 55%	H−1→L+0 17%	H−0→L+1 19%	
(*E*)-DAC3	π → π*	2.660 (466.21)	2.253	0.994	H−0→L+0 55%	H−1→L+0 19%	H−0→L+1 19%	
(*E*)-DAC4	π → π*	2.507 (494.51)	2.091	0.992	H−0→L+0 54%	H−1→L+0 20%	H−0→L+1 18%	
(*Z*)-DAC1	n → π*	2.450 (496.05)	0.330	0.532	H−0→L+0 17%	H−1→L+0 11%	H−0→L+1 27%	H−2→L+1 21%
	π → π*	3.156 (392.87)	0.907	0.876	H−0→L+0 22%	H−1→L+0 36%	H−1→L+1 10%	
(*Z*)-DAC2	n → π*	2.490 (497.98)	0.382	0.585	H−0→L+0 16%	H−1→L+0 14%	H−0→L+1 25%	H−1→L+1 14%
	π → π*	3.121 (397.31)	0.785	0.836	H−0→L+0 25%	H−1→L+0 27%	H−0→L+1 13%	
(*Z*)-DAC3	n → π*	2.487 (498.55)	0.358	0.561	H−0→L+0 17%	H−1→L+0 13%	H−0→L+1 26%	H−1→L+1 11%
	π → π*	3.134 (395.60)	0.823	0.850	H−0→L+0 24%	H−1→L+0 30%	H−0→L+1 12%	
(*Z*)-DAC4	n → π*	2.453 (505.38)	0.457	0.651	H−0→L+0 17%	H−0→L+1 20%	H−2→L+0 17%	H−2→L+1 22%
	π → π*	3.234 (383.38)	0.890	0.871	H−0→L+0 11%	H−0→L+1 43%	H−3→L+0 27%	

## Supplementary Materials

The following are available online at http://www.mdpi.com/2079-4991/9/1/119/s1: Figure S1: Conjugative interaction energies (ΔE^(2)^, in kcal/mol) between the π and π* orbitals as a function of the d_1_, d_2_, and d_4_ bond distance for (a) *trans* dyes and (b) *cis* dyes; Figure S2: Plots of excitation energy vs. distance d_1_ and d_4_ for (a) *trans* dyes and (b) *cis* dyes; Figure S3: Plots of the NBO charges of π-spacer moiety vs. distance d_1_ and d_4_ for (a) *trans* dyes and (b) *cis* dyes; Figure S4: Plots of the NBO charges of donor moiety vs. distance d_1_, d_2_, and d_4_ for (a) *trans* dyes and (b) *cis* dyes; Figure S5: Plots of the NBO charges of acceptor moiety vs. distance d_1_, d_2_, and d_4_ for (a) *trans* dyes and (b) *cis* dyes; Table S1: Conjugative interaction energies (ΔE^(2)^, in kcal/mol) between the π and π* orbitals in the azobenzene-based dyes from the second-order perturbation theory analysis within NBO analysis.

## References

[1] Hardin B.E., Snaith H.J., McGehee M.D. (2012). The renaissance of dye-sensitized solar cells. Nat. Photonics.

[2] Zhang S., Yang X., Numata Y., Han L. (2013). Highly efficient dye-sensitized solar cells: Progress and future challenges. Energy Environ. Sci..

[3] Fakharuddin A., Jose R., Brown T.M., Fabregat-Santiago F., Bisquert J. (2014). A perspective on the production of dye-sensitized solar modules. Energy Environ. Sci..

[4] Liang M., Chen J. (2013). Arylamine organic dyes for dye-sensitized solar cells. Chem. Soc. Rev..

[5] Wu Y., Zhu W. (2013). Organic sensitizers from D-π-A to D-A-π-A: Effect of the internal electron-withdrawing units on molecular absorption, energy levels and photovoltaic performances. Chem. Soc. Rev..

[6] Mahmood A. (2016). Triphenylamine based dyes for dye sensitized solar cells: A review. Sol. Energy.

[7] Lee C.-P., Li C.-T., Ho K.-C. (2017). Use of organic materials in dye-sensitized solar cells. Mater. Today.

[8] Martisnovich N., Troisi A. (2011). Theoretical studies of dye-sensitized solar cells: From electronic structure to elementary processes. Energy Environ. Sci..

[9] Labat F., Bahers T.L., Ciofini I., Adamo C. (2012). First-principles modeling of dye-sensitized solar cells: Challenges and perspectives. Acc. Chem. Res..

[10] Preat J., Michaux C., Jacquemin D., Perpete E.A. (2009). Enhanced efficiency of organic dye-sensitized solar cells: Triphenylamine derivatives. J. Phys. Chem. C.

[11] Peng B., Yang S., Li L., Cheng F., Chen J. (2010). A density functional theory and time-dependent density functional theory investigation on the anchor comparison of triarylamine-based dyes. J. Chem. Phys..

[12] Al-Eid M., Lim S., Park K.-W., Fitzpatrick B., Han C.-H., Kwak K., Hong J., Cooke G. (2014). Facile synthesis of metal-free organic dyes featuring a thienylethynyl spacer for dye sensitized solar cell. Dyes Pigment..

[13] Merino E., Ribagorda M. (2012). Control over molecular motion using the *cis-trans* photoisomerization of the azo group. Beilstein J. Org. Chem..

[14] Wagner-Wysiecka E., Lukasik N., Biernat J.F., Luboch E. (2018). Azo group(s) in selected macrocyclic compounds. J. Incl. Phenom. Macrocycl. Chem..

[15] Zhang L., Cole J.M. (2014). TiO_2_-assisted photoisomerization of azo dyes using self-assembled monolayers: Case study on *para*-methyl red towards solar-cell applications. ACS Appl. Mater. Interfaces.

[16] Kakiage K., Aoyama Y., Yamamura M., Yano T., Unno M., Kyomen T., Hanaya M. (2014). A novel alkoxysilyl azobenzene dye photosensitizer with alkylamino group for dye-sensitized solar cells. Silicon.

[17] Ezema C.G., Nwanya A.C., Ezema B.E., Patil B.H., Bulakbe R.N., Ukoha P.O., Lokhande C.D., Maaza M., Ezema F.I. (2016). Photo-electrochemical studies of chemically deposited nanocrystalline meso-porous n-type TiO_2_ thin films for dye-sensitized solar cell (DSSC) using simple synthesized azo dye. Appl. Phys. A.

[18] Chiu K.Y., Tran T.T.H., Chang S.H., Yang T.-F., Su Y.O. (2017). A new series of azobenzene-bridged metal-free organic dyes and application on DSSC. Dyes Pigment..

[19] Ye Y., Pang J., Zhou X., Huang J. (2016). Understanding the torsion effects on optical properties of azobenzene derivatives. Comput. Theor. Chem..

[20] Novir S.B., Hashemianzadeh S.M. (2017). Quantum chemical investigation of structural and electronic properties of trans- and cis-structures of some azo dyes for dye-sensitized solar cells. Comput. Theor. Chem..

[21] Frisch M.J., Trucks G.W., Schlegel H.B., Scuseria G.E., Robb M.A., Cheeseman J.R., Scalmani G., Barone V., Petersson G.A., Nakatsuji H. (2009). Gaussian 09, Revision C.01.

[22] Naumov V.A., Samdal S., Naumov A.V., Gundersen S., Volden H.V. (2005). Molecular Structure of Triphenylamine in the Gas Phase. Russ. J. Gen. Chem..

[23] Preat J., Jacquemin D., Michaux C., Perpète E.A. (2010). Improvement of the efficiency of thiophene-bridged compounds for dye-sensitized solar cells. Chem. Phys..

[24] Jacquemin D., Wathelet V., Perpète E.A., Adamo C. (2009). Extensive TD-DFT Benchmark: Singlet-Excited States of Organic Molecules. J. Chem. Theory Comput..

[25] Berardo E., Hu H.-S., van Dam H.J.J., Shevlin S.A., Woodley S.M., Kowalski K., Zwijnenburg M.A. (2014). Describing Excited State Relaxation and Localization in TiO_2_ Nanoparticles Using TD-DFT. J. Chem. Theory Comput..

[26] Yin T., Zhao Z.X., Zhang H.X. (2018). Theoretical Study of Substituent and Charge Effects on the Thermal Cis→ Trans isomerization of Ortho-fluoroazobenzenes Photoswitches. Org. Electron..

[27] Tomasi J., Mennucci B., Cammi R. (2005). Quantum Mechanical Continuum Solvation Models. Chem. Rev..

[28] Yang J., Ganesan P., Teuscher J., Moehl T., Kim Y.J., Yi C., Comte P., Pei K., Holcombe T.W., Nazeeruddin M.K. (2014). Influence of the donor size in D–π–A organic dyes for dye-sensitized solar cells. J. Am. Chem. Soc..

[29] Boschloo G., Hagfeldt A. (2003). Photoinduced absorption spectroscopy of dye-sensitized nanostructured TiO_2_. Chem. Phys. Lett..

[30] Tigreros A., Ortiz A., Insuasty B. (2014). Effect of π-conjugated linkage on photophysical properties: Acetylene linker as the better connection group for highly solvatochromic probes. Dyes Pigment..

[31] Mo Y., Lin Z., Wu W., Zhang Q. (1996). Bond-distorted orbitals and effects of hybridization and resonance on C–C bond lengths. J. Phys. Chem..

[32] Kreglewski M. (1989). The geometry and inversion-internal rotation potential function of methylamine. J. Mol. Spectrosc..

[33] Pearson R., Lovas F.J. (1977). Microwave spectrum and molecular structure of methylenimine (CH_2_NH). J. Chem. Phys..

[34] Bouwstra J.A., Schouten A., Kroon J. (1983). Structural studies of the system trans-azobenzene/trans-stilbene. I. A reinvestigation of the disorder in the crystal structure of trans-azobenzene, C_12_H_10_N_2_. Acta Crystallogr. Sect. C.

[35] Zhang J., Zheng H., Zhang T., Wu M. (2009). Theoretical Study for High-Energy-Density Compounds Derived from Cyclophosphazene. IV. DFT Studies on 1,1-Diamino-3,3,5,5,7,7-hexaazidocyclotetraphosphazene and Its Isomers. Int. J. Mol. Sci..

[36] Santhanamoorthi N., Lo C.M., Jiang J.C. (2013). Molecular Design of Porphyrins for Dye-Sensitized Solar Cells: A DFT/TDDFT Study. J. Phys. Chem. Lett..

[37] Song J., Xu J. (2013). Density functional theory study on D-p-A-type organic dyes containing different electron-donors for dye-sensitized solar cells. Bull. Korean Chem. Soc..

[38] Martin R.L. (2003). Natural transition orbitals. J. Chem. Phys..

[39] Campbell W.M., Jolley K.W., Wagner P., Wagner K., Walsh P.J., Gordon K.C., Schmidt-Mende L., Nazeeruddin M.K., Wang Q., Gratzel M. (2007). Highly Efficient Porphyrin Sensitizers for Dye-Sensitized Solar Cells. J. Phys. Chem. C.

[40] Sun C., Li Y., Song P., Ma F. (2016). An Experimental and Theoretical Investigation of the Electronic Structures and Photoelectrical Properties of Ethyl Red and Carminic Acid for DSSC Application. Materials.

[41] Angelis F.D., Fantacci S., Selloni A., Gratzel M., Nazeeruddin M.K. (2007). Influence of the sensitizer adsorption mode on the open-circuit potential of dye-sensitized solar cells. Nano Lett..

[42] Georgiev A., Bubev E., Dimov D., Yancheva D., Zhivkov I., Krajcovic J., Vala M., Weiter M., Machkova M. (2017). Synthesis, Structure, Spectral properties and DFT Quantum Chemical Calculations of 4-aminoazobenzene Dyes. Effect of Intramolecular Hydrogen Bonding on photoisomerization. Spectrochim. Acta Part A.

[43] Zhang C.-R., Liu L., Zhe J.-W., Jin N.-Z., Ma Y., Yuan L.-H., Zhang M.-L., Wu Y.-Z., Liu Z.-J., Chen H.-S. (2013). The Role of the Conjugate Bridge in Electronic Structures and Related Properties of Tetrahydroquinoline for Dye Sensitized Solar Cells. Int. J. Mol. Sci..

[44] Seo D., Park K.W., Kim J., Hong J., Kwak K. (2016). DFT computational investigation of tuning the electron donating ability in metal-free organic dyes featuring a thienylethynyl spacer for dye sensitized solar cells. Comput. Theor. Chem..

