# Supplementary material for: Computational Investigation of Tuning the Electron-Donating Ability in Metal-Free Organic Dyes Featuring an Azobenzene Spacer for Dye-Sensitized Solar Cells
Source: Nanomaterials (Basel). 2019 Jan 18;9(1):119. doi: 10.3390/nano9010119 (PMC6359366; doi:10.3390/nano9010119)
Supplement: Supplementary file 1 [file nanomaterials-09-00119-s001.pdf]

Computational investigation of tuning the electron donating ability in metal-free organic dyes featuring an azobenzene spacer for dye-sensitized solar cells

Md Al Mamunur Rashid [1,$], Dini Hayati [2,$], Kyungwon Kwak [1,*] and Jongin Hong [2,*]

[1] Center for Molecular Spectroscopy and Dynamics, Institute for Basic Science (IBS), & Department of Chemistry, Korea University, Seoul 02841, Republic of Korea; ndcmamun@korea.ac.kr (M.A.M.R.)
[2] Department of Chemistry, Chung-Ang University, Seoul 06974, Republic of Korea; dinihayati300194@gmail.com (D.H.)
* Correspondence: kkwak@korea.ac.kr (K.K.); hongj@cau.ac.kr (J.H.)

Table S1. Conjugative interaction energies ($\Delta E^{(2)}$, in kcal/mol) between the π and π^* orbitals in the azo-benzene based dyes from the second-order perturbation theory analysis within NBO analysis.

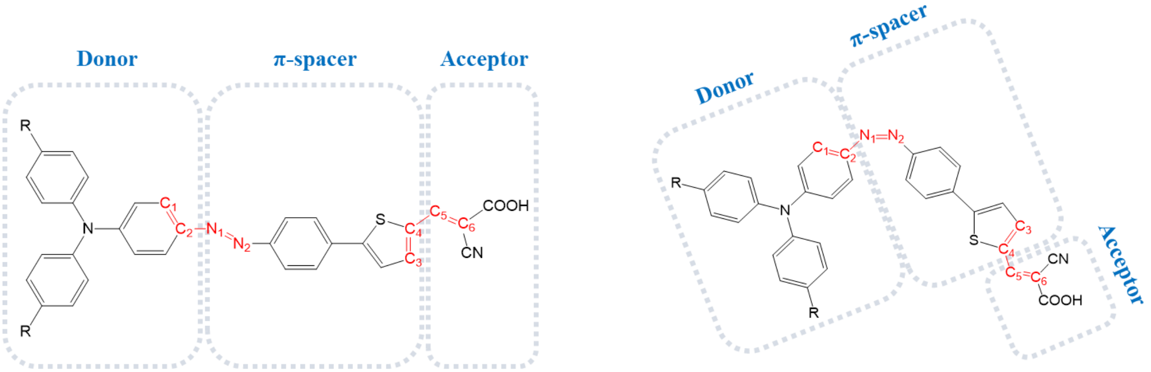

Dyes	Donor orbital (π)	Acceptor orbital (π^*)	$\Delta E^{(2)}$ [kcal mol^{-1}]	E_{acc}-E_{don} [a.u]	F(acc, don) [a,u]
(E)-DAC1	$\pi(C_1{\equiv}C_2)$	$\pi^*(N_1{=}N_2)$	24.06	0.23	0.069
	$\pi(C_3{=}C_4)$	$\pi^*(C_5{=}C_6)$	23.25	0.30	0.075
(E)-DAC2	$\pi(C_1{\equiv}C_2)$	$\pi^*(N_1{=}N_2)$	24.55	0.23	0.069
	$\pi(C_3{=}C_4)$	$\pi^*(C_5{=}C_6)$	23.36	0.30	0.075
(E)-DAC3	$\pi(C_1{\equiv}C_2)$	$\pi^*(N_1{=}N_2)$	25.13	0.23	0.070
	$\pi(C_3{=}C_4)$	$\pi^*(C_5{=}C_6)$	23.49	0.30	0.075
(E)-DAC4	$\pi(C_1{\equiv}C_2)$	$\pi^*(N_1{=}N_2)$	26.27	0.22	0.071
	$\pi(C_3{=}C_4)$	$\pi^*(C_5{=}C_6)$	23.77	0.30	0.075
(Z)-DAC1	$\pi(C_1{\equiv}C_2)$	$\pi^*(N_1{=}N_2)$	15.53	0.24	0.057
	$\pi(C_3{=}C_4)$	$\pi^*(C_5{=}C_6)$	23.37	0.30	0.075
(Z)-DAC2	$\pi(C_1{\equiv}C_2)$	$\pi^*(N_1{=}N_2)$	16.26	0.24	0.058
	$\pi(C_3{=}C_4)$	$\pi^*(C_5{=}C_6)$	23.48	0.30	0.075
(Z)-DAC3	$\pi(C_1{\equiv}C_2)$	$\pi^*(N_1{=}N_2)$	17.61	0.24	0.061
	$\pi(C_3{=}C_4)$	$\pi^*(C_5{=}C_6)$	23.62	0.30	0.075
(Z)-DAC4	$\pi(C_1{\equiv}C_2)$	$\pi^*(N_1{=}N_2)$	19.48	0.23	0.063
	$\pi(C_3{=}C_4)$	$\pi^*(C_5{=}C_6)$	24.83	0.29	0.077

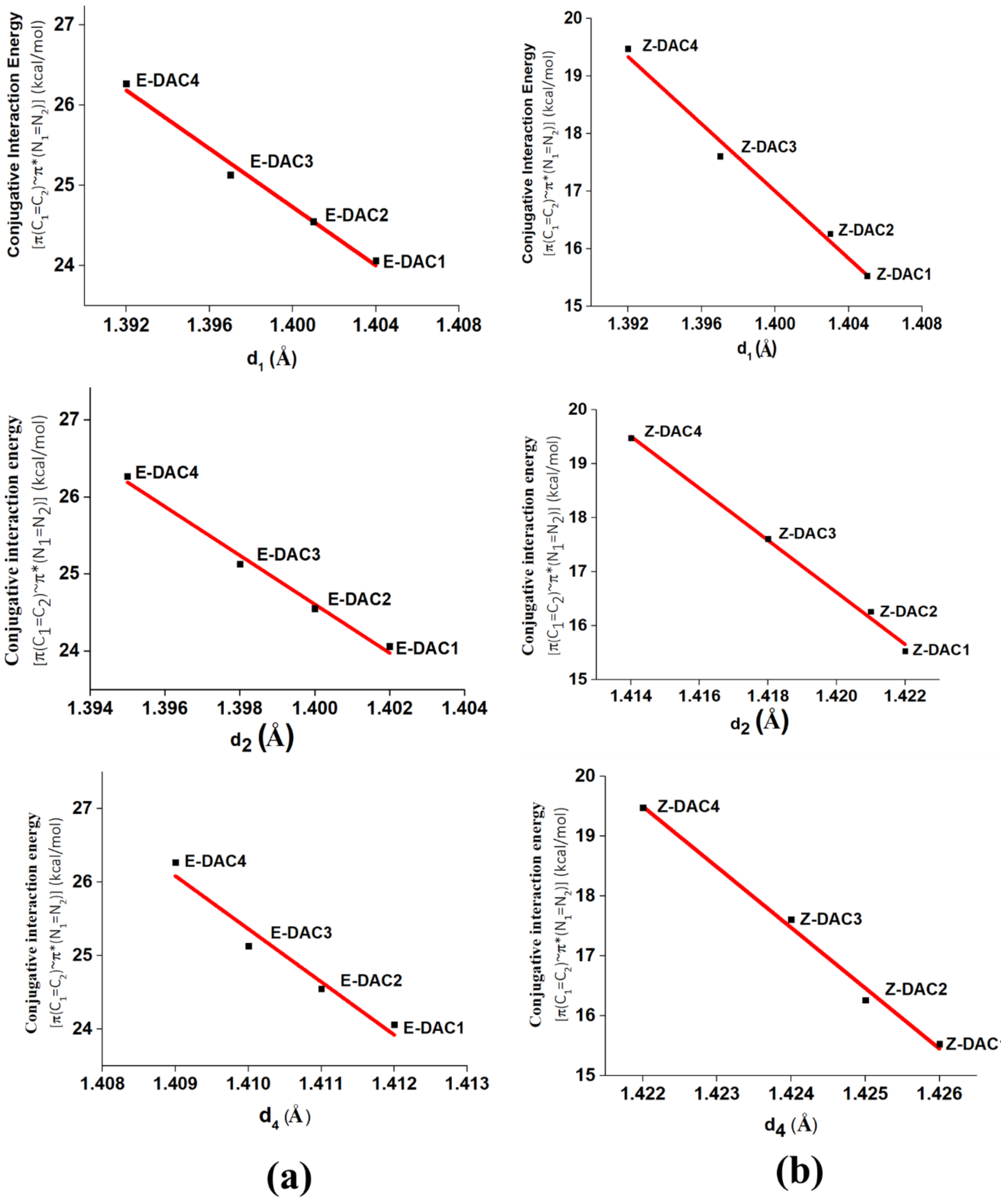

Figure S1 Conjugative interaction energies ($\Delta E^{(2)}$, in kcal/mol) between the π and π* orbitals as a function of the d_1, d_2, and d_4 bond distance for (a) trans dyes, and (b) cis dyes.

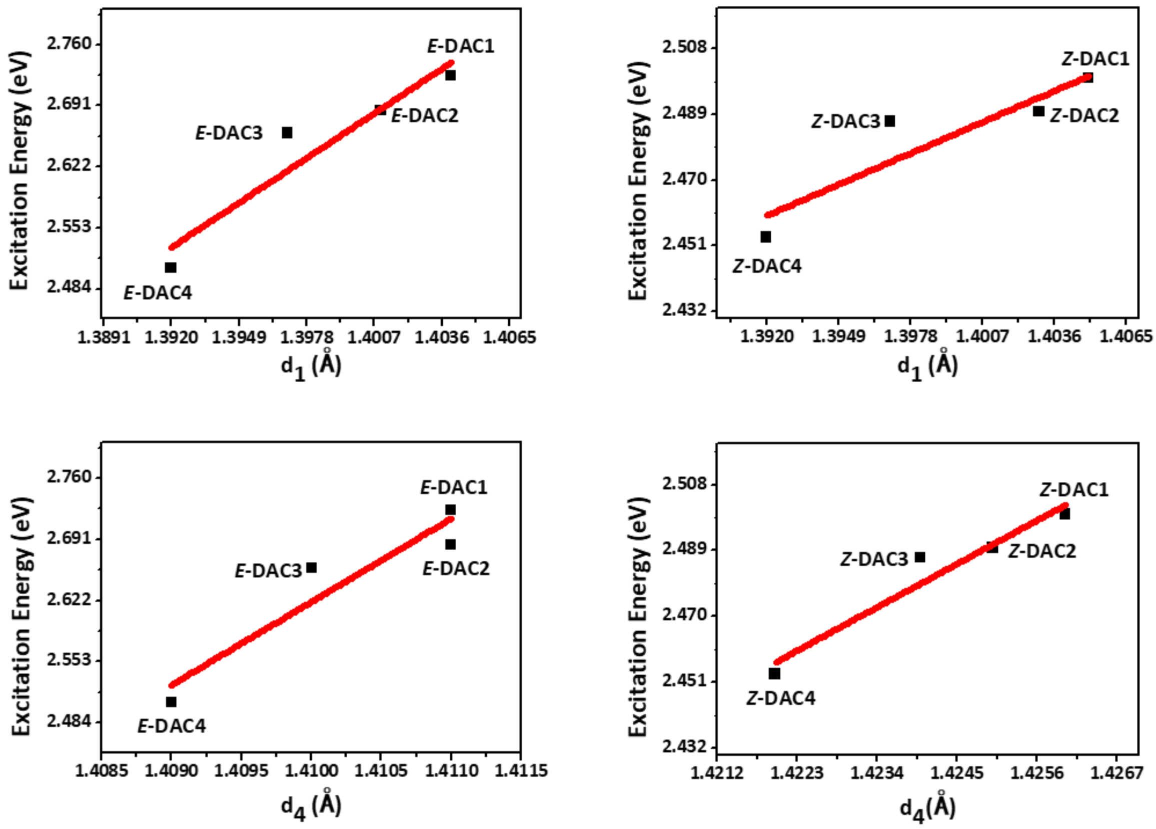

(a) **(b)**

Figure S2 Plots of Excitation energy vs distance d_1, and d_4 for (a) trans dyes and (b) cis dyes.

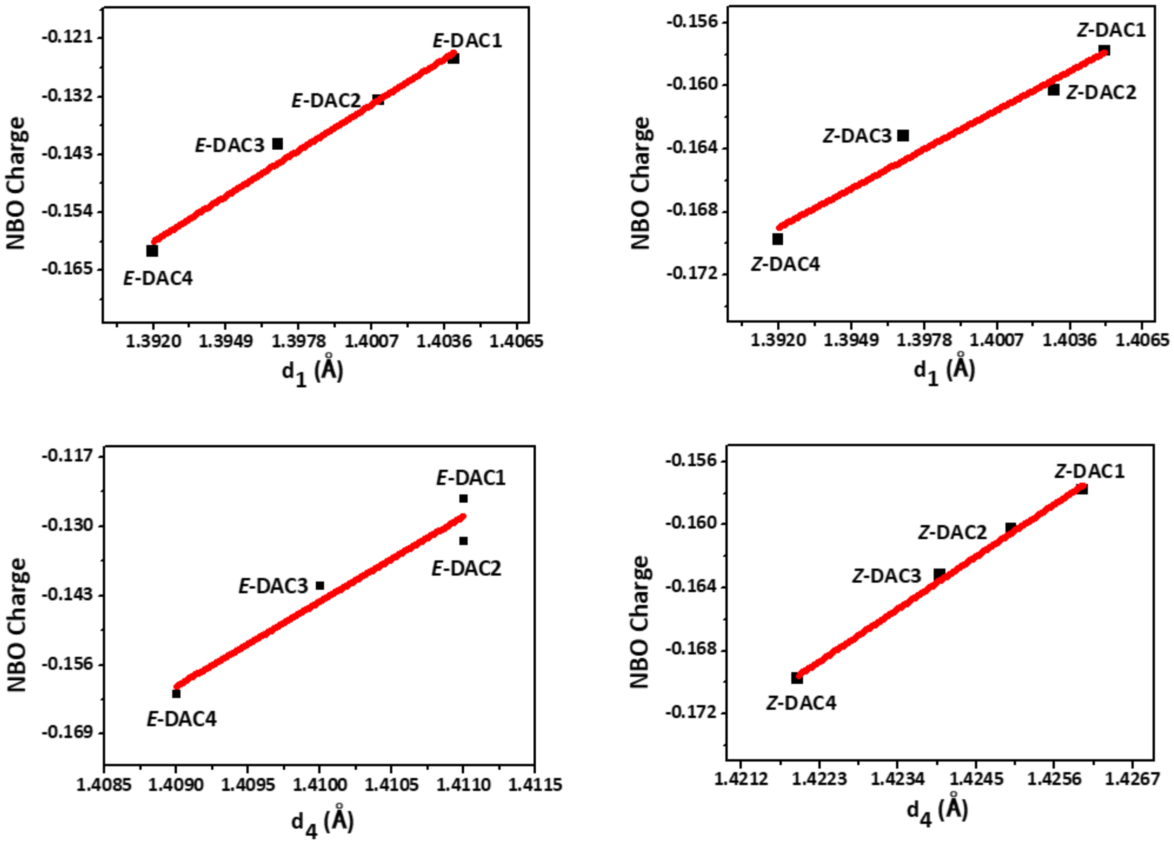

(a) **(b)**

Figure S3 Plots of the NBO charges of π-spacer moiety vs distance d_1, and d_4 for (a) trans dyes and (b) cis dyes.

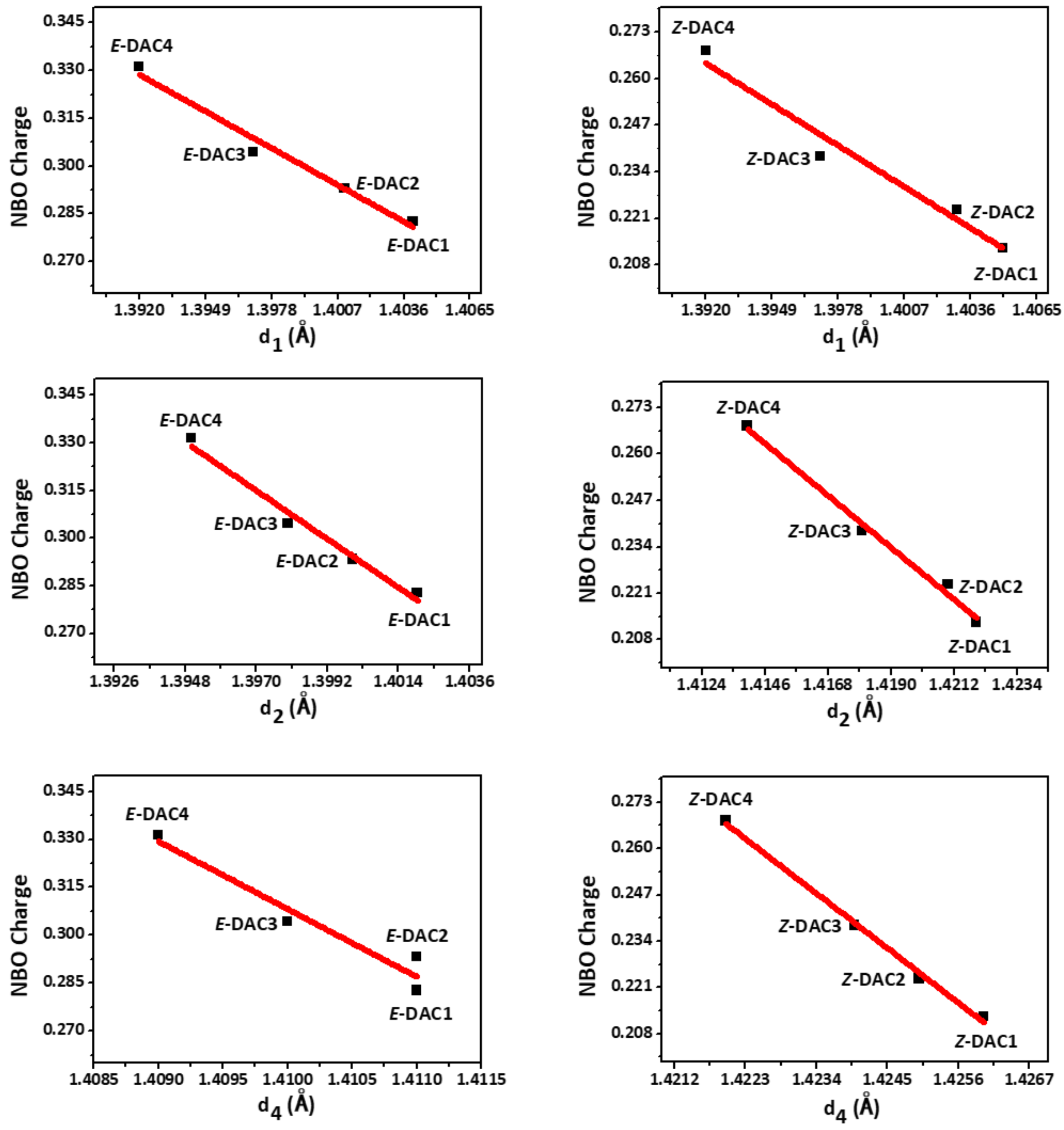

(a) **(b)**

Figure S4 Plots of the NBO charges of donor moiety vs distance d_1, d_2 and d_4 for (a) trans dyes and (b) cis dyes.

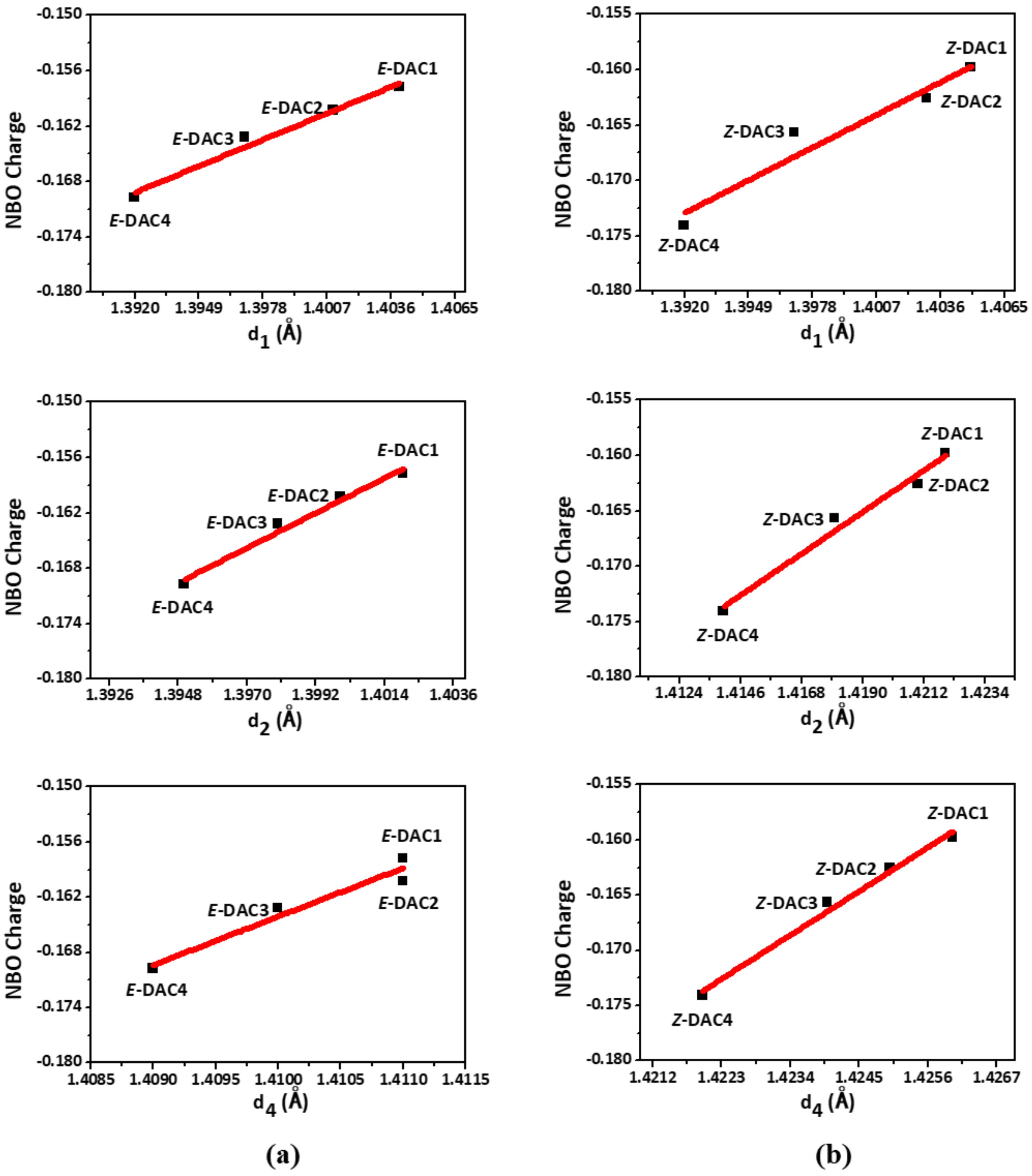

Figure S5 Plots of the NBO charges of acceptor moiety vs distance d_1, d_2 and d_4 for (a) trans dyes and (b) cis dyes.
